# Progress and inequalities in infant and young child feeding practices in India between 2006 and 2016

**DOI:** 10.1111/mcn.12663

**Published:** 2018-11-29

**Authors:** Phuong Hong Nguyen, Rasmi Avula, Derek Headey, Lan Mai Tran, Marie T. Ruel, Purnima Menon

**Affiliations:** ^1^ Poverty, Health and Nutrition Division International Food Policy Research Institute Washington, DC USA; ^2^ Alive & Thrive FHI 360 Washington, DC USA

**Keywords:** breastfeeding, complementary feeding, India, inequity, infant and young child feeding practices

## Abstract

Limited evidence exists on socio‐economic status (SES) inequalities in infant and young child feeding (IYCF) in India. We examine trends and changes in inequalities for IYCF practices over 2006–2016 and identify factors that may explain differences in IYCF across SES groups. We use data from the 2015–2016 and 2005–2006 National Family Health Surveys (*n* = 112,133 children < 24 months). We constructed SES quintiles (Q) and assessed inequalities using concentration and slope indices. We applied path analyses to examine the relationship between SES inequalities, intermediate determinants, and IYCF. Breastfeeding improved significantly over 2006–2016: from 23% to 42% for early initiation of breastfeeding (EIBF) and 46% to 55% for exclusive breastfeeding (EBF). Minimum dietary diversity (MDD) improved modestly (15% to 21%), but adequate diet did not change (~9%). Large SES gaps (Q5–Q1) were found for EIBF (8–17%) and EBF (−15% to −10%) in 2006; these gaps closed in 2016. The most inequitable practices in 2006 were MDD and iron‐rich foods (Q5 ~ 2–4 times higher than Q1); these gaps narrowed in 2016, but levels are low across SES groups. Factors along the path from SES inequalities to IYCF practices included health and nutrition services, information access, maternal education, number of children < 5 years, and urban/rural residence. The improvements in breastfeeding and narrowing of equity gaps in IYCF practices in India are significant achievements. However, ensuring the health and well‐being of India's large birth cohort will require more efforts to further improve breastfeeding, and concerted actions to address all aspects of complementary feeding across SES quintiles.

**Key messages**

- This study uses two of India's nationally representative household surveys conducted 10 years apart (2006 and 2016) to examine trends and changes in inequalities for IYCF practices overtime and identify factors that may explain differences in IYCF across socio‐economic groups.
- Our findings highlight significant improvements in breastfeeding practices and closing of equity gaps in EBF, mainly due to improvements in Q5. Although the equity gaps in complementary feeding practices also narrowed, complementary feeding shows slow progress and poor practices across all segments of society.
- These results call for special efforts to further improve breastfeeding, and concerted actions to address all aspects of complementary feeding across SES quintiles.

## 1 INTRODUCTION

Appropriate nutrition during early life, including adequate infant and young child feeding (IYCF) practices, is essential for optimal growth and development. Despite strong technical guidance and recommendations for age‐appropriate IYCF practices for children below 2 years (Pan American Health Organization, 2003; World Health Organization [WHO], 2008), global progress on these practices has been slow. Socio‐economic inequalities in malnutrition and access to effective nutrition and health services continue to exist throughout the world (Black et al., 2013; Victora et al., 2018; Victora & Somers, 2015). Children living in resource‐poor settings are generally at a greater disadvantage than their wealthier counterparts with respect to living conditions, access to preventive care and complementary feeding practices (Barros, Victora, Scherpbier, & Gwatkin, 2010), although poorer households are often more likely to breastfeed and to do so for longer periods of time. Reducing inequality—which is at the heart of a wide range of Sustainable Development Goals—is therefore critical for achieving nutrition goals (Global Nutrition Report, 2017).

India, a country with 1.3 billion population, contributes to two thirds of the global burden of undernutrition and ranks high among the most unequal countries in the world on consumption expenditure, income, and wealth (Himanshu, 2018; World Bank, 2010). In addition to socio‐economic factors, geographic inequalities such as state‐specific or urban and rural residence disparities influence nutrition outcomes and their determinants, including access to health services and preventive and curative interventions. For example, neonatal mortality is higher among low‐income compared with high‐income states (Million Death Study Collaborators et al., 2010). The prevalence of stunting also varies widely across states and across rural and urban areas, with higher burden among the poor, especially among the urban poor (Kanjilal, Mazumdar, Mukherjee, & Rahman, 2010). In India, inequalities in socio‐economic status (SES) and place of residence are particularly evident for access and use of antenatal care (ANC) services, which favour the rich and urban populations as seen in the higher average number of ANC visits and higher quality of ANC among these population groups (Viegas Andrade, Noronha, Singh, Rodrigues, & Padmadas, 2012).

Although there is ample documentation of socio‐economic and regional inequalities in maternal and child health in India, there is limited evidence on inequalities in IYCF practices. A study using the 2005–2006 India National Family Health Survey (NFHS) reported that high wealth index and urban residence were associated with lower prevalence of exclusive breastfeeding (EBF) (Patel et al., 2012). Similarly, evidence from 36 developing countries, including India, showed that although complementary feeding practices are generally better in urban areas compared with rural areas, breastfeeding (BF) practices are consistently worse (Smith, Ruel, & Ndiaye, 2005). However, these studies examined inequalities for urban/rural and wealth quintile separately and focused on relative ratios, rather than more robust measures that take into account the cumulative population wealth distribution. Hence, a more in‐depth assessment of inequalities in IYCF practices, considering intersectionality (López & Gadsden, 2016) between wealth and residence, is essential for strategic investment, and targeting and planning of interventions to close the equity gap.

In this study, we address this knowledge gap by focusing on three objectives: (a) examine trends in IYCF practices between 2006 and 2016, (b) assess the changes in absolute and relative socio‐economic inequalities in IYCF practices in both rural and urban areas, and (c) identify factors associated with socio‐economic inequalities that explain differences in IYCF practices.

## 2 METHODS

### 2.1 Data sources

This paper uses nationally representative data from the India 2005–2006 NFHS‐3 (International Institute for Population Sciences [IIPS], 2007) and the 2015–2016 NFHS‐4 (IIPS, 2017), conducted by the IIPS, under the stewardship of the Ministry of Health and Family Welfare (MoHFW), Government of India. These surveys contain extensive data on population, health, and nutrition, and a range of underlying determinants. The NFHS‐3 survey consisted of data from 109,041 sample households and was representative at the state level. The NFHS‐4 survey is unique in being the first national survey to be representative at both state and district levels, gathering data from 601,509 households. These surveys are also representative at urban/rural levels.

Both surveys used a stratified two‐stage sample design. The first stage involved selection of primary sampling units, which were the villages in rural areas and the Census Enumeration Blocks in urban areas. Within each stratum, villages or blocks were selected from the sampling frame with probability proportional to population size. The second stage involved the random selection of 22 households with systematic sampling method from each primary sampling unit where a complete household mapping and listing operation was conducted prior to the main survey. Each survey contained four well‐structured separate datasets for households, men, women aged 15–49 years, and children under 5 years of age. Because this paper focuses on IYCF practices, analyses were restricted to the mother–child dyads in which the child was under 24 months old (*n* = 18,474 in NFHS‐3 and 93,659 in NFHS‐4).

### 2.2 Variables

#### 2.2.1 Outcome variables

IYCF practices were assessed using the standard WHO indicators (WHO, 2008), on the basis of the maternal recall of all foods and liquids given to children in the 24 hr prior to the survey. The two key BF indicators were (a) early initiation of BF (EIBF—defined as the proportion of infants who were put to the breast within 1 hr of birth) and (b) EBF (defined as the proportion of infants 0–6 months of age who were fed only breast milk). In order to examine the BF pattern, we categorized BF status into exclusive (as defined above), BF + plain water, BF + nonmilk liquid, BF + other milk, BF + formula, BF + solid/semisolid foods, and no BF (United Nations Children's Fund [UNICEF], in press).

We constructed five complementary feeding indicators for children 6–23 months old: (a) timely introduction of complementary foods (defined as the proportion of infants aged 6–8 months who received solid, semisolid, or soft foods in the previous 24 hr), (b) minimum dietary diversity (defined as children who consumed foods from four or more food groups out of seven food groups in the previous 24 hr), (c) minimum meal frequency as appropriate for age, (d) minimum acceptable diet (defined as children who the both minimum dietary diversity and age‐appropriate minimum meal frequency), and (e) consumption of iron‐rich food (WHO, 2008). We also reported specific foods and the total number of food groups consumed by the target child in the previous day.

#### 2.2.2 Variables used for equity analyses

A household SES index was constructed using the principal component analysis method, extracting from multiple variables including house and land ownership, housing structure, access to services (electricity, gas, water, and sanitation services), and ownership of 17 assets (car, motorbike, bicycle, television, radio, computer, refrigerator, watch, mobile phone, fan, bed, mattress, table, chair, press cooker, sewing machine, and water pump) and livestock (cow, goat, chicken; Filmer & Pritchett, 2001; Vyas & Kumaranayake, 2006). Principal component analysis was applied to both rounds of data to construct a consistent SES index. The first principal component explained 65% of the variance and was used to divide household SES into quintiles, stratified by urban and rural areas; the lowest quintile (Q1) represented the poorest 20% of the pooled population, and the highest quintile (Q5) represented the richest 20%.

#### 2.2.3 Potential factors associated with changes in IYCF practices

The selection of potential determinants of changes in IYCF practices was guided by the conceptual framework, particularly the UNICEF (1990) and Lancet Nutrition Series (Black et al., 2013). In this paper, we used four groups: (a) household factors, (b) maternal factors, (c) child factors, and (d) health and nutrition services.

Household‐level variables included area of residence (rural, urban), religion, scheduled caste/tribal (designated groups of historically disadvantaged people in India), number of children < 5 years, and household SES. Maternal characteristics included age, age at first birth (at 18 years or older), education, occupation, and access to information. Mothers' occupation was only available for ~20% of sample in 2016 and, therefore, was excluded from the analyses. Access to information was measured by the proportion of mothers reporting to watch TV, listen to radio, or read newspaper daily. Child factors included child age, sex, and birth order.

We examined several nutrition and health services across the continuum of care (pregnancy, delivery, and early childhood). Services received during pregnancy included at least four ANC visits, iron and folic acid (IFA) consumption (at least 100 IFA tablets during the last pregnancy), neonatal tetanus protection, deworming, weight monitoring, and BF counselling by front‐line workers. Indicators related to services during delivery included skilled birth attendance and caesarean section. Institutional delivery was not examined because it was highly correlated with skilled birth attendance. Indicators related to early childhood services included full immunization, paediatric IFA and vitamin A supplementation, and deworming. A score of 1 was given for each service that mothers received during pregnancy or early childhood, and the average score for each period was used in the analyses. We also examined Integrated Child Development Services (ICDS) services, specifically food supplementation for pregnant or lactating mothers and children. Due to the age specificity of IYCF practices, nutrition and health services during pregnancy were used for modelling BF practices, and nutrition and health services during early childhood were used for modelling complementary feeding practices.

### 2.3 Data analysis

We used several complementary methods to analyse the data. First, we used graphical methods to document changes in the age profile of IYCF patterns and regression models to examine changes in IYCF indicators over time. We also tested for differences in various determinants between 2006 and 2016 using linear regression models (for continuous variables) and logistic regression models (for categorical variables), adjusting for standard errors for the cluster sampling design and sampling weights used in the survey.

Second, we estimated the absolute gap (difference between the wealthiest and poorest quintiles [Q5 − Q1]) and the slope index of inequality (SII) to explore absolute SES inequalities in IYCF practices for rural and urban separately. We calculated relative gap (Q5/Q1 ratios) and the concentration index (CIX) to examine relative SES inequalities (O'Donnell, Doorslaer, Wagstaff, & Lindelow, 2008; O'Donnell, O'Neill, Van Ourti, & Walsh, 2016). Although absolute and relative gaps are simple indices that allow to convey results to nontechnical audiences and public health experts, these measures do not capture the intermediate population groups (e.g., Q2–Q4) and are sensitive to changes in the number of individuals in each stratification category (Barros & Victora, 2013). The SII and CIX account for the entire SES distribution of the sample by wealth score (Barros & Victora, 2013), with SII expressed in percentage points and CIX as a range between −1 and +1 (with 0 representing equality between the rich and the poor, and positive values indicating a prorich distribution). CIX values are multiplied by 100 for presentation. The SII was estimated by using a regression approach, and the CIX was calculated using analogous approach by ranking individuals according to SES position. These two measures were also used to assess whether inequalities increased or declined over time.

Third, we explored the underlying factors associated with recent changes in SES inequalities by examining quintile‐specific changes between 2006 and 2016 for these factors. Finally, we conducted bivariate and multivariable regression analyses and found that associations between SES and IYCF practices were highly significant in the bivariate models but became insignificant in multivariable models, suggesting potential mediation effects. Therefore, we applied path analyses to assess the complex relationships between SES status and underlying factors of IYCF and with three key IYCF outcomes (EIBF, EBF, and minimum dietary diversity). We estimated the models separately for each survey round and pooled (combining the data from both rounds). Given that differences between the separate and pooled models were minimal, we only report the pooled results.

All analyses were performed using Stata Version 15.1. All regression models were adjusted for standard errors for the cluster sampling design and sampling weights used in the survey.

## 3 RESULTS

The characteristics of the study population by survey round are presented in Table 1. There were several significant economic and social changes from 2006 to 2016 in India, especially in relation to household SES, urbanization, women's education, age at first marriage, and access to information. We also observed significant and in many cases large improvements over time in access, use, and coverage of nutrition and health services across the continuum of care. For example, the percentage of mothers receiving four ANC visits increased by ~15 percentage points, consumption of IFA supplements during pregnancy doubled (from 15% to 30%), and BF counselling during pregnancy increased by more than threefold (from 13% to 42%). There were also remarkable improvements in coverage of child immunization (from 35% to 50%), vitamin A (from 22% to 60%) and paediatric IFA supplementation (from 5% to 26%), and deworming (8% to 28%). There was a large increase (close to threefold) in the percentage of women and children receiving food supplementation during pregnancy, lactation, or early childhood.

**Table 1:** Characteristics of the study sample, by survey year

Characteristic	2006	2016
	*n* = 18,474	*n* = 93,659
	Mean/per cent (95% CI)	Mean/per cent (95% CI)
Household		
No. of children < 5, *n*	1.59 [1.57, 1.61]	1.51*** [1.51, 1.52]
SES index,^a^ *n*	−0.53 [−0.56, −0.49]	0.03*** [0.02, 0.05]
Religion (Hindu), %	78.88 [77.06, 80.70]	78.57 [77.85, 79.29]
Religion (Muslim), %	16.28 [14.51, 18.05]	16.70 [16.01, 17.38]
Scheduled caste/tribe, %	70.99 [69.40, 72.57]	76.41*** [75.80, 77.01]
Reside in urban areas, %	23.23 [21.21, 25.25]	26.93** [25.99, 27.88]
Mothers		
Age, years	25.05 [24.91, 25.19]	25.65*** [25.59, 25.70]
Education, years	4.29 [4.12, 4.46]	6.66*** [6.60, 6.73]
Age at first birth ≤ 18, %	43.93 [42.45, 45.41]	21.26*** [20.78, 21.75]
Working outside the home^b^	32.78 [30.36, 33.20]	16.46*** [15.59, 17.33]
Access to information, %	39.85 [38.32, 41.39]	54.63*** [54.02, 55.25]
Child		
Gender (male), %	53.07 [51.91, 54.22]	52.66 [52.12, 53.20]
Age, months	14.43 [14.30, 14.55]	14.46 [14.40, 14.51]
Birth order, *n*	2.69 [2.63, 2.75]	2.18 [2.17, 2.20]
Nutrition and health services		
During pregnancy		
At least 4 ANC visits, %	35.55 [33.81, 37.28]	50.18*** [49.49, 50.87]
Consumed 100+ IFA, %	15.28 [14.24, 16.32]	30.09*** [29.45, 30.73]
Weighed at least once, %	48.30 [46.26, 50.35]	75.39*** [74.83, 75.95]
Neonatal tetanus protection, %	79.33 [77.89, 80.78]	87.87*** [87.46, 88.29]
Deworming, %	3.82 [3.35, 4.30]	17.85*** [17.32, 18.38]
Breastfeeding counselling, %	13.16 [12.13, 14.19]	41.90*** [41.23, 42.57]
Delivery		
Institutional birth, %	39.89 [38.04, 41.74]	82.32*** [81.81, 82.83]
Skilled birth attendance, %	47.94 [46.09, 49.80]	84.26*** [83.80, 84.72]
Caesarean section, %	8.91 [8.16, 9.66]	18.64*** [18.10, 19.18]
Early childhood		
Full immunization, %	35.00 [33.53, 36.47]	50.26*** [49.67, 50.86]
Vitamin A supplementation, %	21.79 [20.58, 23.01]	60.34*** [59.72, 60.96]
Paediatric IFA, %	4.81 [4.27, 5.35]	25.75*** [25.16, 26.34]
Paediatric deworming, %	7.74 [6.91, 8.57]	27.52*** [26.92, 28.11]
Food supplementation		
For pregnant women, %	20.15 [18.88, 21.42]	53.73*** [53.09, 54.36]
For lactating mothers, %	15.38 [14.28, 16.49]	48.51*** [47.88, 49.13]
For children, %	18.84 [17.65, 20.04]	52.59*** [51.97, 53.20]

*Note*. ANC: antenatal care; IFA: iron and folic acid; SES: socio‐economic status.

a The SES index was obtained from the principal component analysis, and it has *M* = 0 and *SD* = 1.

b Data only available for subsample of women in 2016 (*n* = 16,466).

*** *p* < 0.001.

** *p* < 0.01.

* *p* < 0.05.

**Figure 1:**
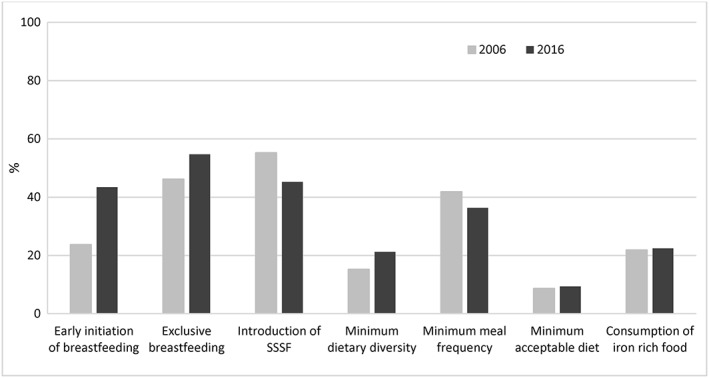
Changes in infant and young child feeding practices between 2006 and 2016. SSSF, semisolid and solid food

**Figure 2:**
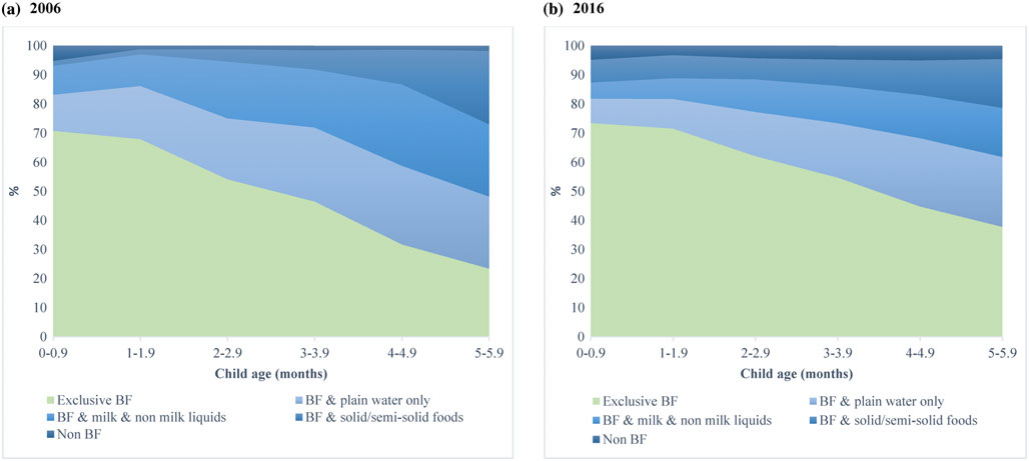
Changes in breastfeeding (BF) patterns between (a) 2006 and (b) 2016

Between 2006 and 2016, there was a significant improvement in BF practices. EIBF nearly doubled (from 23% to 42%), and EBF increased from 46% to 55% (Figure 1), mainly due to reduction in feeding water, milk, or nonmilk liquids (Figure 2). However, worryingly, nearly 5% of infants in all quintiles were not breastfed at all in 2016. Prelacteal feeding in the first 3 days after birth was very common in 2006, practised by 57% in 2006, but reduced sharply to 21% in 2016 (Table S1). The largest reductions in commonly fed prelacteals were fresh milk (32% to 13%), honey (14% to 3%), sugar water (11% to 2%), and plain water (9% to 3%).

Complementary feeding is of a major concern, with nearly a 10‐percentage‐point decline in timely introduction of semisolid foods (55% to 45%) and in minimum meal frequency (42% to 36%) between 2006 and 2016 (Figure 1). Minimum acceptable diet (~9%) is extremely low and did not change over time. Minimum dietary diversity increased slightly (15% to 21%), mainly due to increased consumption of eggs (5% to 15%), vitamin A‐rich fruit and vegetables (35% to 40%), and other fruits and vegetables (15% to 24%; Figure S1).

**Figure 3:**
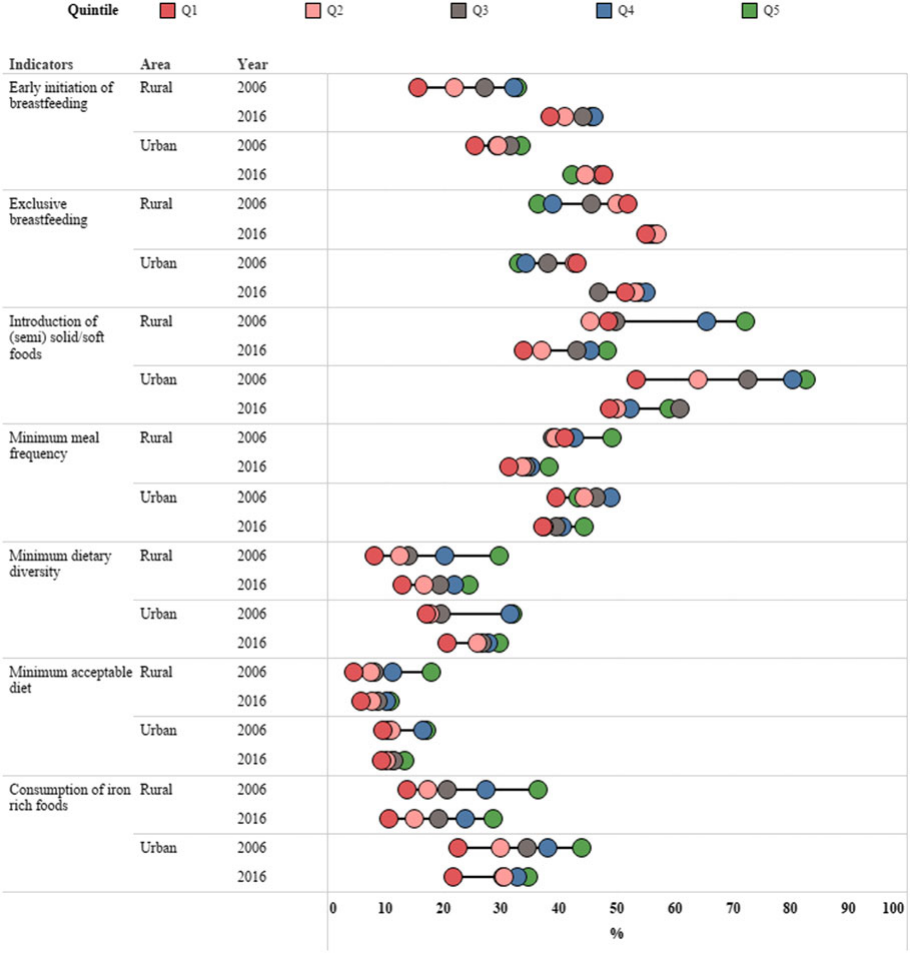
Inequality trends in infant and young child feeding practices between 2006 and 2016, by socio‐economic status quintile and rural/urban residence

Table 2 presents estimates of absolute and relative inequality indices for key IYCF practices as well as the SII and CIX by rural and urban areas and by survey year. Figure 3 visualizes these IYCF practices in the equiplots where each dot represents the prevalence of a given IYCF practice for a quintile subgroup and the distance between the dots is the gap between the quintiles. Most IYCF practices (except for EBF) exhibited prorich inequality patterns where recommended practices were higher among the rich than among the poor within urban and rural populations. Gaps between Q5 and Q1 for EIBF were larger for rural (17%) compared with urban areas (8%) in 2006 but narrower in 2016 in both areas. EBF showed a different pattern where in 2006 EBF was higher among the lower SES compared with the higher SES (Q5–Q1: −15% in rural and −10% in urban or SII: −19% and −12%, respectively), but these gaps were much smaller in 2016, mostly due to improvements in EBF in Q5, especially in rural areas.

**Table 2:** Inequity gaps in infant and young child feeding practices between 2006 and 2016, by SES quintile and rural/urban residence

Indicators	Area	Year	Q1	Q5	Q5–Q1	SII	Q5/Q1	CIX
Early initiation of breastfeeding	Rural	2006	15.75	32.95	17.20	17.42***	2.09	3.57***
		2016	38.39	45.59	7.20	9.26***	1.19	1.58***
	Urban	2006	25.63	33.54	7.91	10.72***	1.31	1.37***
		2016	47.67	42.19	−5.48	−2.08	0.89	−0.88***
Exclusive breastfeeding	Rural	2006	51.92	36.43	−15.49	−19.44***	0.70	2.85***
		2016	54.97	55.19	0.22	−2.23	1.00	−0.09
	Urban	2006	43.12	33.08	−10.04	−11.75*	0.77	1.64*
		2016	51.55	53.81	2.26	4.02	1.04	0.45
Timely introduction of SSSF	Rural	2006	48.52	72.21	23.69	27.30***	1.49	3.50***
		2016	33.86	48.34	14.48	15.62***	1.43	3.00***
	Urban	2006	53.40	82.75	29.35	36.83***	1.55	6.48***
		2016	48.84	59.01	10.17	15.64***	1.21	1.48**
Minimum meal frequency	Rural	2006	41.07	49.15	8.08	12.27***	1.20	1.53***
		2016	31.37	38.20	6.83	6.18***	1.22	1.32***
	Urban	2006	39.56	43.38	3.82	27.08***	1.10	3.81***
		2016	37.29	44.27	6.98	10.02***	1.19	1.48***
Minimum dietary diversity	Rural	2006	8.26	29.85	21.59	19.78***	3.61	3.14***
		2016	12.97	24.36	11.39	12.45***	1.88	2.24***
	Urban	2006	17.20	32.06	14.86	21.78***	1.86	3.27***
		2016	20.76	29.71	8.95	11.20***	1.43	1.49***
Minimum acceptable diet	Rural	2006	4.53	18.03	13.50	12.91***	3.98	1.98***
		2016	5.84	10.86	5.02	5.31***	1.86	1.00***
	Urban	2006	9.54	17.09	7.55	13.83***	1.79	1.67***
		2016	9.41	13.43	4.02	5.68***	1.43	0.76***
Consumption of iron‐rich foods	Rural	2006	13.78	36.26	22.48	20.42***	2.63	3.31***
		2016	10.73	28.69	17.96	20.52***	2.67	3.65***
	Urban	2006	22.62	43.94	21.32	25.67***	1.94	4.22***
		2016	21.67	34.81	13.14	17.93***	1.61	2.09***

*Note*. CIX: concentration index; Q: quintile; SES: socio‐economic status; SSSF: semisolid and solid food; SII: slope of inequity index.

*** *p* < 0.001.

** *p* < 0.01.

* *p* < 0.05.

Absolute gaps between the richest and poorest quintiles were wide for timely introduction of solid/semisolid foods in 2006 using both the Q5–Q1 (24% for rural and 29% for urban areas) and the SII summary measures (27% for rural and 37% for urban areas). In 2016, there was a decline in the timely initiation of complementary foods across quintiles and in both urban and rural areas (with a greater magnitude of decline in rural areas). There was also a narrowing of the gaps between quintiles in both urban and rural areas. Similar declines in the proportion of children with minimum meal frequency between 2006 and 2016 were also observed, but reductions in the wealth inequality gap were smaller than for timely initiation of complementary foods. Minimum dietary diversity and consumption of iron‐rich foods also had a large gap in 2006 where children belonging to Q5 consumed two to four times more than did those in the Q1, and the gap between the two extreme quintiles was ~20%. These gaps narrowed in 2016, but levels are low among all quintiles and lower in 2016 compared with 2006 for the highest SES quintile in both urban and rural areas. The proportion of children with minimum acceptable diet was very low (<20% even among Q5 in both urban and rural areas) and declined between 2006 and 2016 for the highest two SES quintiles. In terms of specific food groups, the largest wealth gaps were for dairy consumption in both urban and rural areas (Figure S2). The magnitude of the gap was reduced in 2016 for both areas, and the percentage of children consuming dairy was higher in urban areas for all SES quintiles. The consumption of food groups other than starchy staples was low for all segments of society (<10% for flesh foods, <20% for eggs, <30% for legumes, and <40% for vitamin A‐rich fruits and vegetables) and did not show much difference between rural–urban groups or the 2006 and 2016 surveys.

**Figure 4:**
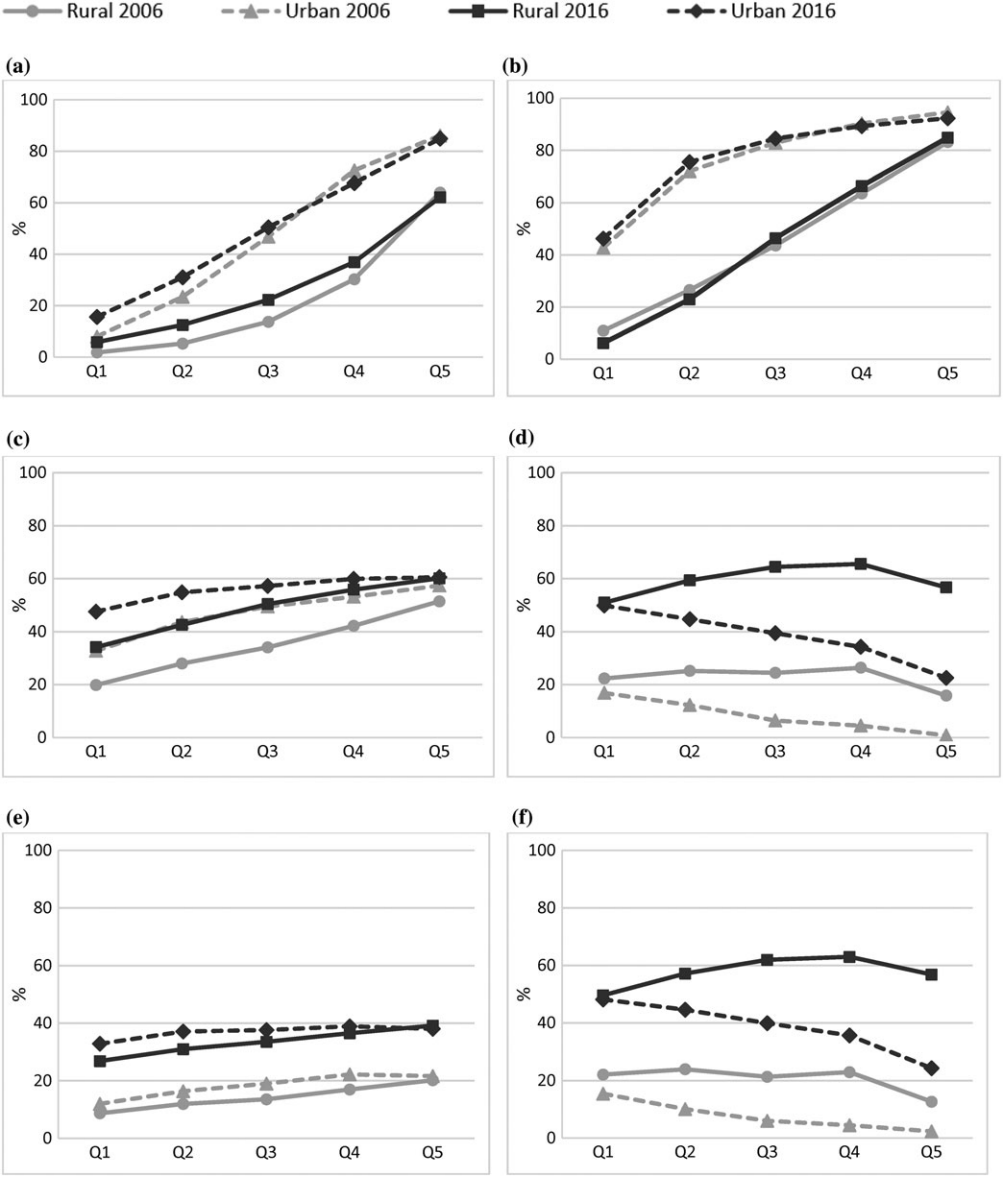
Selected potential determinants of infant and young child feeding practices, by socio‐economic status quintile, rural/urban residence, and survey year. (a) Mother has at least 10 years of schooling. (b) Access to information every day. (c) Nutrition and health services during pregnancy. (d) Food supplementation for pregnant women. (e) Nutrition and health services during childhood. (f) Food supplementation for children

**Figure 5:**
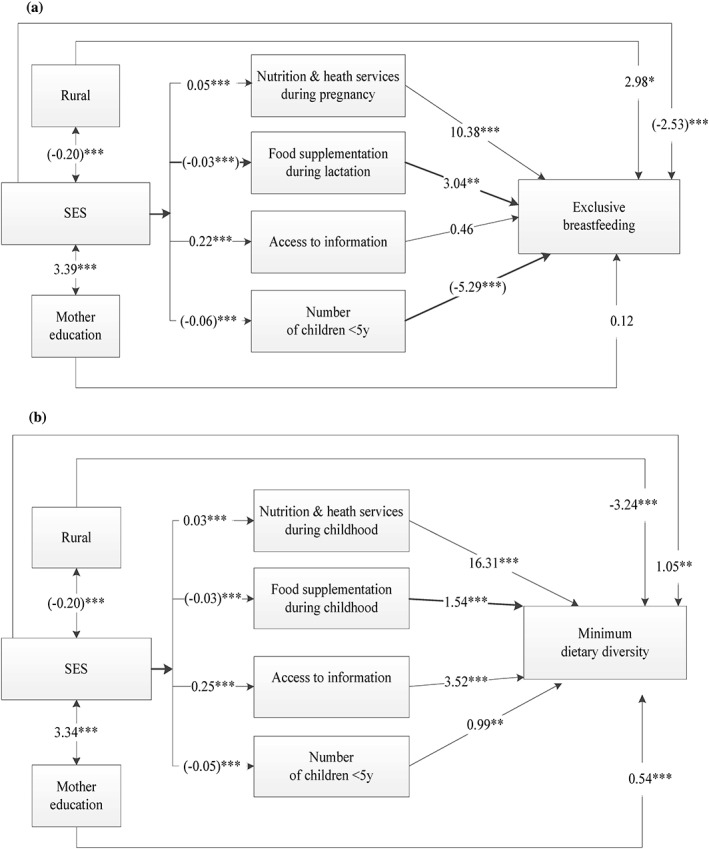
Path models for early initiation of breastfeeding, exclusive breastfeeding, and minimum dietary diversity. (a) Exclusive breastfeeding. (b) Minimum dietary diversity. SES, socio‐economic status

Figure 4 shows the wealth and urban/rural differentials for a select number of determinants of IYCF practices at the two survey times. For most of the determinants (except food supplementation), the Q5 quintile showed higher coverage, and urban areas performed better than did rural areas. Results from path analyses pooling the 2006 and 2016 surveys (Figure 5 and Figure S3) show significant positive associations between SES quintiles and mother's education (*β* = 3.4), access to information (*β* = 0.22 to 0.25), and nutrition and health services (*β* = 0.03 to 0.05). These factors, in turn, are positively associated with IYCF practices. For example, compared with women who did not use nutrition and health services during pregnancy, those who used the services were 29% more likely to initiate early BF and 10% more likely to exclusively breastfeed. Similarly, children who used the services were 16% more likely to achieve minimum diet diversity than were those who did not. The relationship between SES quintiles, food supplementation, and IYCF practices was complex. Because ICDS services are more widely used by the poor, food supplementation was higher in lower quintiles and in rural areas, but receiving food supplementation was positively associated with IYCF practices (*β* = 5.1 for EIBF, 3.0 for EBF, and 1.5 for minimum dietary diversity).

## 4 DISCUSSION

Our analysis of India's nationally representative household surveys conducted 10 years apart (2006 and 2016) showed significant improvements in BF practices, especially among the wealthier quintile and a closing of the equity gap in EBF. Although a narrowing of the equity gaps was also found in complementary feeding practices, this remains a critical concern, showing deterioration over time in timely introduction of solid/semisolid foods, slow progress on minimum dietary diversity and minimum acceptable diets, and overall poor complementary feeding practices across all segments of society. SES appears to be an important driver of both BF and complementary feeding practices operating through a complex series of sociodemographic factors, greater access and use of health and nutrition services, and access to information pathways.

Although programme and policy strategies in India are well aligned with global guidance on IYCF and provide a vision for intervention scale‐up (Avula, Oddo, Kadiyala, & Menon, 2017), progress in improving IYCF practices has been mixed, and several practices remain suboptimal. Prior research using the 2005–2006 NFHS documented poor IYCF practices in India and strong associations between most IYCF practices and SES status and maternal education (Patel et al., 2010). Strikingly, our results suggest that progress since 2006 has largely been limited to BF practices, and especially so among the wealthiest groups (Q5), whereas the proportion of children introduced to complementary foods in a timely fashion and achieving minimum meal frequency declined, and dietary diversity improved only marginally. We find indicative evidence that improvements in BF were driven by improved access to, and use of, health and nutrition services during pregnancy for the lowest SES quintiles. This suggests that targeting of the services to poorer socio‐economic groups improved over time. Among the wealthier quintile (Q5), however, use of health services did not increase in urban areas and increased only marginally in rural areas between 2006 and 2016, suggesting that quality, rather than greater use of counselling on EBF, may have improved over time. The closing of the equity gap in EBF is a remarkable story, but still only about half of all children 0–6 months of age in our urban and rural samples were exclusively breastfed at the time of the survey in 2016.

Thus, strengthening health and nutrition programmes in India to support optimal IYCF practices through counselling is important, especially for BF and those aspects of complementary feeding that are amenable to behaviour change even when resources are limited (for instance, timely introduction of semisolid and solid food). For dietary diversity, it is likely that economic and cultural factors are major constraints to adoption of optimal practices. Carefully designed, culturally sensitive counselling and awareness raising are essential. Young children tend to be fed household diets in many low‐ and middle‐income countries (Nguyen et al., 2013), probably for economic reasons. Purchasing special nutrient‐rich foods for young children may be prohibitively expensive for poor households and is not justified. Recent research shows that animal‐sourced foods (ASFs), for example, are expensive in low‐income countries, with calories from ASFs being typically five to 10 times more expensive than calories from staple cereals (Headey, Alderman, Maitra, & Rao, 2017). Although some ASFs such as dairy and poultry products are cheaper in India than in most low‐ or middle‐income countries (because India is now a major producer of these foods), it does not mean that they are within reach for the poorest segments of the population. Cash transfers could be one of the mechanisms to improve household diet. An evidence review of cash transfers vis‐à‐vis food transfers suggests that unconditional and conditional cash transfers improve household food consumption and dietary diversity (Alderman, 2016). Evidence that this translates into improvements in young children's diets, however, is scant. The NFHS data suggest that around one third of all Indian women report never consuming meat or eggs, and 11% report never consuming dairy, indicating a set of truly vegetarian families where promotion of ASFs is not an option. However, among the remaining two thirds of families, removing additional constraints (economic and cultural), preventing the feeding of ASF products to young children could contribute to filling some of the critical gaps in complementary feeding practices that exist among this age group.

The worsening of complementary feeding practices in India occurred even in the context of increasing use of health services and of the ICDS programme. We found that receiving food supplements from the ICDS was associated with improved IYCF practices. It is possible that additional efforts to improve the composition and uptake of the complementary food supplements in the ICDS programme, or to include additional ASFs, could help increase children's dietary diversity through public provisioning of such foods. Some state governments have been proactive in introducing eggs into the ICDS programme (Khera, 2015), and there may be further scope to scale up the use of a variety of ASFs in ICDS and other programmes. The ICDS and health platforms in India, both of which have high coverage in many states, could also play a much stronger role in supporting complementary feeding behaviours by strengthening the behaviour change communication and community mobilization components of the programme. The evidence on the impact of strategies deployed within or alongside the ICDS to address IYCF behaviours is limited (Avula et al., 2017), but ample evidence now exists of group‐based and individual counselling strategies in India that have been successful at improving complementary feeding practices (Nair et al., 2017; Vazir et al., 2013). Adapting these for scale‐up through the ICDS platform, or in collaboration with the ICDS, could help close the gaps in IYCF knowledge and practices. However, given that these programme platforms are generally propoor, it is also imperative to examine ways to improve the reach of IYCF counselling even among the upper quintiles, where IYCF practices are still poor and have even worsened over time. This will require additional strategies such as working with private providers/paediatricians, the media, and other platforms.

Our findings on inequality in child feeding are consistent with more conventional economic analyses of inequality in India. Historically, household consumption data suggested that inequality in India was not especially high by international standards, but the more recent switch to income‐based inequality measures revealed that economic inequality in India was among the highest in the world (Himanshu, 2018). India's rapid economic growth has also coincided with rising inequality, although growth in consumption among the bottom 40% has been reasonably rapid, at 3.2% per year over 2004–2011, compared with 3.7% for the population as a whole (World Bank, 2016). The past decade has also seen the introduction or scale‐up of major poverty reduction efforts, including reforms to the Public Distribution Scheme, National Rural Employment Guarantee Scheme, and significant shifts in occupations and urbanization. However, both secular and economic trends and the quality of poverty reduction programmes vary markedly across states. Despite the efforts of government to improve access to health care among women and children, especially for the underserved population (MoHFW, 2005), we still found substantial SES inequalities in use of nutrition and health care services in both urban and rural areas, a finding consistent with a previous review (Sanneving, Trygg, Saxena, Mavalankar, & Thomsen, 2013). In addition, although these services improved in the last decade, the coverage remains suboptimal, such as BF counselling during pregnancy (42%) or interventions during early childhood (25–60%). Further improvement in access to nutrition and health services along the continuum of care, particularly for the poor, can have potential impacts on IYCF practices.

Our study has several unique strengths. We use two rounds of large nationally representative datasets to explore changes in IYCF practices for different SES groups. The NFHS has several strengths in this regard, including consistency in a wide range of key indicators, national and state representativeness, and scope for measuring SES through well‐established asset indices (Filmer & Pritchett, 2001). For inequity analyses, we used both simple methods (absolute inequity Q5–Q1 and relative inequity Q5/Q1) that can be easily conveyed to lay audiences and advocate to policymakers, and the advanced methods (SII and CIX) that account for the overall frequency of the outcomes that have changed markedly over time in our analyses. Results from different analyses were consistent and complemented each other, confirming the decline in inequalities in IYCF over the last 10 years. The pathway analyses also shed light on the different mechanisms by which SES may affect IYCF practices and is a novel application to this particular issue.

This study has some limitations. The measurement of IYCF practices could be prone to bias from maternal recall or social desirability in reporting. However, there is some evidence that the EBF indicator is accurate (Moore et al., 2007), and complementary feeding indicators were widely used in programme monitoring and evaluations. The NFHS contains rich information on maternal and child health and nutrition, but it is more limited in terms of sociocultural data and does not include any information on nutrition knowledge or exposure to counselling interventions. Thus, the dataset does not have the needed information to examine the influences of these factors on IYCF practices. Finally, the sample size in NFHS‐4 is much larger than that in NFHS‐3; it is possible that this might influence inequality metrics. To overcome that concern, however, we merged all the asset variables from both survey rounds and used the factor scores from this to create the quintiles from each round of data. That way, we ensure that a Q1 person in 2015 is indeed about as poor as a Q1 person in 2005.

## 5 CONCLUSION

Improvements in BF and the narrowing of equity gaps in IYCF practices, especially in EBF in India, are significant achievements. However, ensuring the health and well‐being of India's large birth cohort will require efforts to further improve BF, and concerted actions to address all aspects of complementary feeding across SES quintiles.

## CONFLICTS OF INTEREST

The authors declare that they have no conflicts of interest.

## CONTRIBUTIONS

PHN conceived the idea, conducted the statistical analyses, and wrote significant sections of the manuscript. RA conducted literature review, reviewed the statistical analyses, and wrote part of the manuscript. LMT conducted the statistical analyses and prepared tables and figures for the manuscript. DH reviewed the statistical analyses, supported interpretation, and wrote part of the manuscript. MTR supported data interpretation and reviewed and edited the manuscript. PM reviewed the statistical analyses, supported data interpretation, and reviewed and edited the manuscript. All authors read and approved the final submitted manuscript.

## Supporting information

Supplementary Table 1: Changes in prelacteal feeding practices during the first 3 days between 2006 and 2016
Supplementary Figure 1: Changes in food group consumption between 2006 and 2016
Supplementary Figure 2: Inequality trends in food group consumption between 2006 and 2016, by SES quintile and rural/urban residence
Supplementary Figure 3: Path models for early initiation of breastfeeding
