# Supplementary material for: Progress and inequalities in infant and young child feeding practices in India between 2006 and 2016
Source: Matern Child Nutr. 2018 Nov 29;14(Suppl 4):e12663. doi: 10.1111/mcn.12663 (PMC6518921; doi:10.1111/mcn.12663)
Supplement: Supplementary file 1 — Supplementary Table 1: Changes in prelacteal feeding practices during the first 3 days between 2006 and 2016 Supplementary Figure 1: Changes in food group consumption between 2006 and 2016 Supplementary Figure 2: Inequality trends in food group consumption between 2006 and 2016, by SES quintile and rural/urban residence Supplementary Figure 3: Path models for early initiation of breastfeeding [file MCN-14-e12663-s001.docx]

**Supplementary Table 1: Changes in prelacteal feeding practices during the first 3 days between 2006 and 2016**

| **Prelacteal feeding** | **2006** | **2016** | **2016-2006** |
| --- | --- | --- | --- |
| Fresh milk | 32.3 | 13.3 | -19 |
| Honey | 13.6 | 2.8 | -10.8 |
| Plain water | 8.7 | 2.6 | -6.1 |
| Sugar water | 10.8 | 2.1 | -8.7 |
| Gripe water | 0.3 | 0.3 | 0 |
| Sugar/salt solution | 2.0 | 0.5 | -1.5 |
| Juice | 0.1 | 0.1 | 0 |
| Formula | 0.7 | 0.7 | 0 |
| Tea | 2.9 | 1.2 | -1.7 |
| Janam | 4.6 | 1.5 | -3.1 |
| Any prelacteal | 57.3 | 20.8 | -36.5 |

**Supplementary Figure 1: Changes in food group consumption between 2006 and 2016**

**Supplementary Figure 2: Inequality trends in food group consumption between 2006 and 2016, by SES quintile and rural/urban residence**


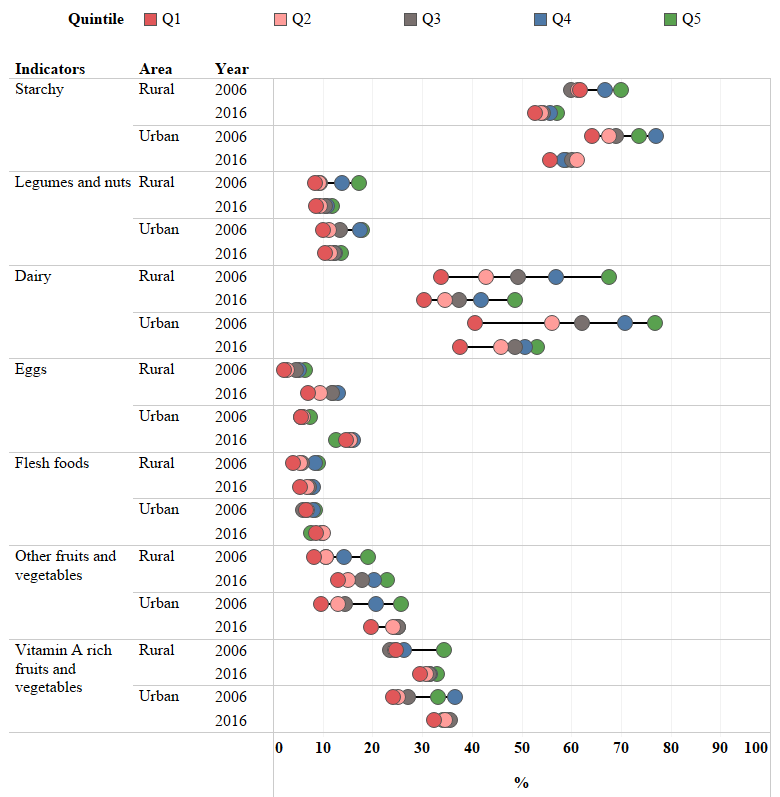


**Supplementary Figure 3: Path models for early initiation of breastfeeding**
